# The early negative bias of social semantics: evidence from behavioral and ERP studies

**DOI:** 10.1186/s40359-023-01286-0

**Published:** 2023-08-26

**Authors:** Xinfang Fan, Qiang Xu, Juan Liu, Hongwei Xing, Liangyu Ning, Qingwei Chen, Yaping Yang

**Affiliations:** 1 Department of Psychology, Ningbo University, Ningbo, China; 2 National Center for International Research on Green Optoelectronics, South China Normal University, Guangzhou, 510006 China; 3 Lab of Light and Physio-Psychological Health, School of Psychology, South China Normal University, Guangzhou, 510631 China; 4 Guangdong Provincial Key Laboratory of Optical Information Materials and Technology, Institute of Electronic Paper Displays, South China Academy of Advanced Optoelectronics, South China Normal University, Guangzhou, 510006 China

**Keywords:** Social semantic information, Trait words, Valence, Negative bias, P2

## Abstract

**Background:**

Compared to nonsocial information, the human brain is more highly sensitive to social information. As a kind of typical social semantic information, the words describing person traits differ from the nonsocial semantic information describing inanimate objects in many ways. It remains to be seen whether the processing of trait words has a valence asymmetric and whether it differs from the processing of nonsocial semantic information in terms of behavioral responses and neural temporal processes.

**Method:**

Taking person and object names as priming stimuli and adjective words only used for describing humans or objects as target stimuli, the present study aimed to investigate the processing characteristics of social and nonsocial semantic information by recording both behavioral and ERP data.

**Results:**

Behavioral results showed that the response times for negative words were significantly slower than those for positive words whether for social or nonsocial semantic information. The accuracy rates of negative words were significantly lower than those of positive words when the targets were social words which is contrary to the nonsocial words. The ERP results indicated that there was a negative bias effect on the processing of both types of information during the whole time course of brain neural activity; that is, the P2, N400, and LPP amplitudes elicited by negative words were larger than those elicited by positive words; However, the negative bias effect of social semantic information started at the early perceptual stage which was significantly earlier than the onset of negative bias of nonsocial semantic information, and was significantly affected by the prime type. In addition, there was a significant semantic conflict N400 effect only for nonsocial semantic information.

**Conclusions:**

Overall, the present study revealed the existence of an early negative bias of social information and provided evidence for the specificity of social information.

**Supplementary Information:**

The online version contains supplementary material available at 10.1186/s40359-023-01286-0.

## Introduction

Compared to nonsocial information, the human brain is more highly sensitive to social information [1, 2]. As a kind of typical social semantic information, the words describing person traits differ from the nonsocial semantic information describing inanimate objects in many ways. Most obviously, these words are exclusive to people, specifically for describing an individual’s personality traits or mental state, and cannot be used to describe inanimate objects. For example, whereas a person maybe described as kind or anxious, such descriptions are rarely used for inanimate objects [3]. Many previous studies based on fMRI technology have demonstrated that person semantic knowledge may be functionally dissociable from other classes of inanimate object semantic knowledge within the brain [3–8]. In addition, it’s worthing noting that the words describing human traits are usually positive or negative, which is reflected in the previous research on personality trait words [9, 10] and social cognitive dimension words [11, 12]. It remains to be seen whether the processing of trait words has a valence asymmetric and whether it differs from the processing of nonsocial semantic information in terms of behavioral responses and neural temporal processes. Therefore, this study sought to use event-related potentials (ERPs) to investigate the processing of behavioral and neural features for social and nonsocial semantic information.

In recent years, some researches based on event-related functional magnetic resonance imaging (fMRI) have demonstrated that person semantic knowledge may be functionally dissociable from other classes of inanimate object semantic knowledge within the brain [3–8]. For example, Mitchell et al. [3] found that a unique pattern of brain activity was associated with person judgments. Specifically, the brain regions implicated in social-cognitive functioning, such as medial prefrontal cortex, superior temporal cortex, intraparietal sulcus, and fusiform gyrus, were generally marked by change from baseline brain activity for person judgments along with significant deactivations for object judgments. This finding was verified by Contreras et al. [5]. Recently, a series of studies found that the anterior temporal lobes were selectively activated by social words (e.g., cautious, mean) compared with nonsocial words (e.g., nutritious, trainable) [4, 6, 8]. A recent study made a clear distinction between the social and valence of words and the results showed that no matter the word valence was positive or negative, the left anterior superior temporal sulcus were significantly more active by social words than by nonsocial words [13].

The studies based on ERPs technology also provided evidence that the processing of social semantic information had specificity by using vocabularies as experimental materials. Wang et al. [14] found that the N400 congruency effect (i.e., the difference between incongruity and congruity) started earlier and had larger amplitude for gender stereotype activation than for lexical semantic activation. Molinaro et al. [15] found that both social stereotype conflict and grammatical conflict can induce the typical N400 effect, but the N400 effect, induced by stereotype conflict, lasted longer and was more widely distributed. In addition, Wang et al. [16] used social information (male/female faces) and nonsocial information (blue/orange squares) as the prime stimuli, and used gender-trait words in blue or orange as the target stimuli, finding that social information induced a larger N270 amplitude than nonsocial information, whether in the explicit or implicit task. Although these studies took the trait words describing people as experimental materials to represent social semantic information, there was lack of the control condition of non-social semantic information. That is, previous researchers didn’t directly explore the differences and similarities of processing between these two types of information.

The processing of negative information has a dominant effect in the fields of attention, memory, individual perception, impression formation, and attribution [17, 18]. Some studies found that people need more time to confirm the color of negative words in the Stroop paradigm, compared to neutral or positive words [19, 20], and it takes longer to process semantic discrimination [21, 22]. Researchers argued that people need to spend more time diverting their attention away from negative stimuli than from neutral or positive stimuli [19, 21, 22]. Han et al. [23] adopted the repetition priming paradigm, taking emotional faces as cue stimuli and facial expressions and emotional words as target stimuli. The results showed that the response times to negative stimuli were longer and the accuracy was lower, whether the target stimuli were facial expressions or emotional words. And negative stimuli induced larger N170 and P2 amplitudes than positive stimuli. Pratto and John [19] argued that negative information has stronger attention-grabbing power, which produces a stronger interference effect in experimental tasks. Other researchers have suggested that people always expect good things, while negative things are unexpected, so the reaction time would be longer [17].

As previously mentioned, the words describing human traits are usually positive or negative. Several studies used human words as experimental materials and manipulated the valence of these words to explore the neural activity characteristics of social semantic information. For example, Zhang et al. [24] selected positive, negative, and neutral words describing people as experimental stimuli, and asked the participants to judge the targets’ valence in a rapid sequence of the visual presentation paradigm. The results showed that negative words first induced a larger P1 amplitude, and then emotional words induced larger N170, EPN, and LPC amplitudes. The LPC amplitude induced by positive words was significantly larger than that induced by negative words. Zhao et al. [25] used the same materials and paradigm, but the participants were asked to determine whether the erected words were presented in a series of inverted false words. The results showed that the negative words induced a larger N170 amplitude, while positive words induced a larger LPP amplitude in this implicit task. Although the human vocabulary selected in the above ERP experiments was limited (six positive, negative, and neutral words each) and the research results weren’t entirely consistent, the two studies confirmed that social semantic information has a negative bias in the early stage of perception (P1, N170). However, due to the lack of the control condition of nonsocial semantic information, it remains to be further invested whether the early negative bias effect is unique to social semantic information.

In sum, the above studies have demonstrated that the social semantic information has specificity. The present study manipulated social semantic information and nonsocial semantic information, and aimed to explore the processing characteristics between the two types of semantic information. Previous studies about social semantic information took descriptive adjectives as experimental materials, thus in order to ensure the nonsocial semantic information match and avoid confusion between the two types of words, that is, to avoid the target words that can be used as both social and nonsocial descriptions. The present study took person and object names as the priming stimuli, while the target stimuli were adjective words only used for describing humans (e.g., generous, lazy) or objects (e.g., delicious, shabby) by drawing on the research of Mitchell et al. [3].

Based on prior findings, the processing of negative information has a dominant effect and the human brain is more sensitive to social information [2]. It was expected that: (1) Response times to negative target words would be slower and the accuracy rate would be lower which may be more significant for social words than nonsocial words. (2) The negative bias effect on social semantic information would occur in the early stage of perception. We knew that the amplitude of the P2 is sensitive to emotionality in words from a number of studies [26–31]. Researchers argued that it reflected rapid attention capture by emotional words [32], and people do rudimentary semantic stimulus classification [33] in the early stage of perception. Therefore, the negative words would elicit larger P2 amplitudes than positive words. (3) The negative bias effect on nonsocial semantic information was expected to occur later. (4) Previous research found that a N400 congruency effect was found for both social semantic and nonsocial semantic information conflict [14, 15]. Hence, there would be a larger N400 when the primes and targets are in conflict.

## Methods

### Participants

Thirty Chinese undergraduate students (19 females and 11 males) were recruited in this experiment in exchange for 30 RMB; they ranged in age from 19 to 22 (*M* = 19.9, *SD* = 1.01). None of the participants was involved in the collection and evaluation of the experimental materials. All participants reported normal or corrected-to-normal vision, had no history of current or past neurological or psychiatric illness, and took no medications known to affect the central nervous system. Five participants were excluded due to excessive artifacts in the electroencephalogram (EEG) data. The research protocol was approved by the ethical committee of Ningbo University.

### Stimuli

The stimuli were Chinese word pairs consisting of a prime word followed by a target word (see Appendix 1).

#### Primes

Following the approach of Mitchell et al. [3] for surveying social and nonsocial information processing, the prime words consisted of 60 person names (e.g., Li Ming, Zhao Yang) and 60 object names (e.g., apple, gloves).

#### Targets

Target words consisted of 30 positive and 30 negative personality traits only used for describing people (e.g., generous, lazy), and 30 positive and 30 negative words only used for describing objects (e.g., delicious, worn out). The 120 target words were determined by the following pretest steps.

Firstly, vocabulary was selected. Specifically, 314 adjective words only used for describing people or inanimate objects were selected from the “Good and Evil Personality Word Glossary” compiled by Jiao et al. [34], the “Chinese Adjective Word System for Fundamental Dimensions of Social Cognition” compiled by Han et al. [12]., and the “Modern Chinese Dictionary”. Secondly, the familiarity and valence of each word were rated. The two types of words were assessed separately by 7-point Likert scales (1 = “very unfamiliar/negative,” 7 = “very familiar/positive”). Thirdly, the final vocabulary was determined. To ensure that the selected target words matched the primes, the selected words were further screened. A total of 30 positive and 30 negative words were selected for each type of word.

Finally, an independent sample *t* test or single-sample *t* test was conducted on the stroke numbers and ratings. The results showed that:

(1) There was no difference in physical properties between the two types of words. No significant difference in stroke numbers were found between the words describing people (*M* = 18.80, *SD* = 4.05) and objects (*M* = 19.67, *SD* = 4.50), *t* (118) = − 1.11, *p* = 0.270.

(2) There were significant differences in the valence between positive and negative words describing people/objects, but there were no significant differences in the valence of positive/negative words for social and nonsocial information. Specifically, the valence scores of positive words describing people (*M* = 5.71, *SD* = 0.30) were significantly higher than those of negative words describing people (*M* = 2.54, *SD* = 0.23), *t* (58) = 46.63, *p* < 0.001; the valence scores of positive words describing objects (*M* = 5.61, *SD* = 0.14) were significantly higher than those of negative words describing people (*M* = 2.59, *SD* = 0.27), *t* (58) = 54.42, *p* < 0.001; but whether the words were positive or negative, there were no significant differences between social and nonsocial information, *t*_positive_(58) = 1.60, *p* = 0.118; *t*_negative_(58) = − 0.83, *p* = 0.411.

(3) All four types of words (positive and negative words describing people, positive and negative words describing objects) had high familiarity (ratings ≥ 6). It is worth noting that the highest scores for familiarity were positive words describing people. The results showed that the familiarity scores of positive words describing people (*M* = 6.29, *SD* = 0.14) were significantly higher than those of negative words describing people (*M* = 6.00, *SD* = 0.17), *t* (58) = 7.18, *p* < 0.001, but there were no significant differences between positive words describing objects (*M* = 6.10, *SD* = 0.13) and negative words describing objects (*M* = 6.02, *SD* = 0.16), *t* (58) = 1.87, *p* = 0.067; the familiarity scores of positive words describing people were significantly higher than those of positive words describing objects, *t* (58) = 5.45, *p* < 0.001, but there were no significant differences between negative words describing people and negative words describing objects, *t* (58) = − 0.61, *p* = 0.545. Thus, there was a high degree of familiarity with positive words describing people. Previous studies on linguistic usage frequency have found that a linguistic positivity bias (LPB) exists for human adjectives [35], meaning that the high level of familiarity of positive words related to people found in the pretest was an inevitable phenomenon. Sears [36] argued that individuals tend to perform positive evaluations when evaluating others in social cognition. It may be that the instinctive avoidance of negative information about people leads to a lower level of familiarity with such words than with positive words, but this does not indicate a lack of familiarity with such information. The result of the single-sample *t* test showed that the familiarity of each category was significantly higher than the median four, *p*s < 0.001.

**Fig. 1:**
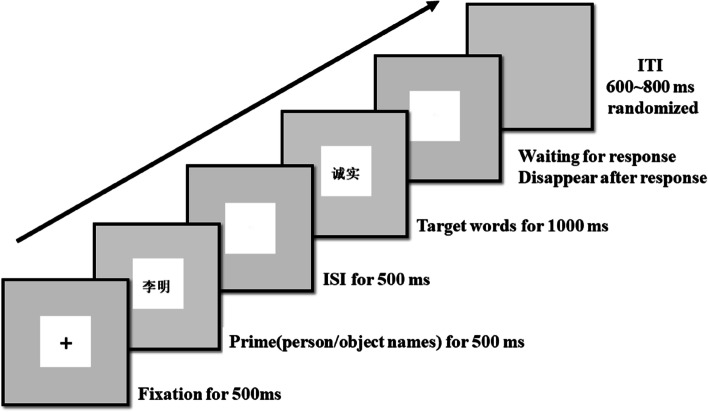
Examples of stimuli presented in the center of a screen with a silver background and the sequences of a trial. In both tasks, a fixation point followed by the presentation of a prime stimulus. After an inter-stimulus interval, the target stimulus was presented. Participants were instructed to verify if the target word was positive or negative. After the ITI randomized between 600 and 800 ms, a next trail began

To sum up, the materials selected in this experiment met the research purpose. All stimuli were presented in the center of a 17-inch screen (resolution 1024 × 768, refresh rate 60 Hz) with a silver background. The font was in regular script (see Fig. 1). Table 1 provides the types of Prime-Targets used in the experiment.

**Table 1:** Types of prime-target (conditions) used in the experiment

Target	Prime	Valence	Prime-target examples	English translation
Social semantic information	Person names	Positive	李明-大度	Li Ming–Generous
		Negative	刘睿-凶狠	Liu Rui–Fierce
	Object names	Positive	水稻-大度	Rice–Generous
		Negative	冰箱-凶狠	Refrigerator–Fierce
Nonsocial semantic information	Person Names	Positive	李明-丰收	Li Ming–Harvest
		Negative	刘睿-老化	Li Ming–Aging
	Object names	Positive	水稻-丰收	Rice–Harvest
		Negative	冰箱-老化	Refrigerator–Aging

### Procedure

The experimental procedure was shown in Fig. 1, each trial began with the appearance of a fixation point for 500 ms. The fixation point was then replaced with a prime stimulus, which remained on the screen for 500 ms. After an inter-stimulus interval of 500 ms (SOA = 1000 ms), the target stimulus was presented for 1000 ms. The screen then went blank and remained blank until the participant responded. The inter-trial interval (ITI) was randomized between 600 and 800 ms. Participants were instructed to verify as quickly and accurately as possible whether the target word was positive or negative by pressing the “积极” (Positive) or “消极” (Negative) button on the response keyboard. The “积极” (Positive) and “消极” (Negative) button were counterbalanced across participants. Participants completed 516 trials, 480 of which constituted the experimental trials (presented in six blocks of 80 trials). The first 32 trials were presented as practice trials to familiarize participants with the procedure. Then there were 4 warm-up trials, followed by the 480 experimental trials. Participants took a break (about 3 to 4 min in length) after each block of 80 trials. Stimuli used in the practice and warm-up trials did not appear in the experimental trials and only data from the experimental trials were analyzed. The experiment lasted for approximately 35 min.

### Electrophysiological recording

The electroencephalogram (EEG) data were recorded continuously using an electrode cap with 64 Ag/AgCl electrodes mounted according to the extended international 10–20 system. Horizontal electro-oculogram (HEOG) and vertical electro-oculogram (VEOG) data were recorded via two pairs of electrodes placed on the bilateral external canthi and left supraorbital and infraorbital areas to monitor eye movements and blinks. Both EEG and EOGs were sampled at 500 Hz, with a bandpass of 0.05–100 Hz using a NeuroScanSynamps2 amplifier. The left mastoid served as a reference during recording and the right mastoid served as a recording electrode. Electrode impedances were kept below 5 kΩ. NeuroScan 4.3 software was used to analyze the data offline, and the data were re-referenced offline to the algebraic average of the left and right mastoids. The raw EEG data were manually previewed to remove artifacts after merging with the behavioral data. Then an automatic eye-movement correction program corrected vertical eye movements and blinks [37]. Afterwards, the EEG data were segmented into epochs of 1200 ms beginning 200 ms prior to the target stimulus onset and each epoch in all electrodes was then corrected to a 200 ms baseline. Segments contaminated with artifacts exceeding an amplitude of ± 90 µV were automatically rejected from the averaging. EEG data from correct trials were averaged separately for each of the eight conditions. After this procedure, averaged ERPs included at least 48 trials for each trial type. Finally, the averaged ERPs were low-pass filtered at 30 Hz (24 dB/Octave).

After careful visual inspection of the grand average waveforms and topographic maps and in accordance with previous studies [24, 25, 38, 39], we focused on the P2, N400, and LPP components, which were significantly influenced by the experimental conditions. The analysis was conducted on ERP amplitudes of clusters of electrodes (regions of interest, ROI). Specifically, for the P2 component the ROI included 14 frontal, frontal-central, and central scalp electrodes (F3, F1, Fz, F2, F4, FC3, FC1, FCz, FC2, FC4, C3, C1, Cz, C2) in the 150–220 ms time-window [23, 40]; for the N400 component the ROI included 14 frontal-central, central, central-parietal, and parietal scalp electrodes (FC1, FCz, FC2, C1, Cz, C2, C4, CP1, CPz, CP2, CP4, P1, Pz, P2) in the 300–400 ms time-window [14, 15, 41]; for the LPP component the ROI included 11 central, central-parietal, parietal, and parietal-occipital scalp electrodes (C3, C1, CZ, CP3, CP1, CPZ, P3, P1, PZ, PO3, POZ) in the 450–600 ms time-window [24, 25, 40].

### Data analysis

The data of response times with inaccurate response and exceeding ± 2*SD* were excluded before the analysis [42]. The RTs, accuracy (ACC) and ERPs results were submitted to a 2(Target: social semantic information vs. nonsocial semantic information) × 2(Prime: person names vs. object names) × 2(Valence: positive vs. negative) repeated-measures ANOVA. For all analyses, if Mauchly’s test showed that the sphericity assumption was violated, Greenhouse–Geisser was applied to correct the degrees of freedom of the *F* test, and Bonferroni corrections were applied to correct the *p*-values for each comparison.

## Results

### Behavioral results

#### Reaction times

The analysis revealed a significant main effect of Target [*F* (1, 24) = 6.08, *p* = 0.021, Partial η^2^ = 0.20], indicating that the response times for social words (*M* = 607.83 ms, *SE* = 23.10) were slower than those for nonsocial words (*M* = 596.70 ms, *SE* = 21.95). The main effect of Valence was significant [*F* (1, 24) = 8.80, *p* = 0.007, Partial η^2^ = 0.27]. Further analysis found that the response times for negative words (*M* = 614.20 ms, *SE* = 24.63) were slower than those for positive words (*M* = 590.33 ms, *SE* = 20.75). Other main effect and interactions were not significant, *p*s > 0.05.

#### Accuracy rates (ACC)

The analysis revealed a significant main effect of Target, *F* (1, 24) = 14.41, *p* = 0.001, Partial η^2^ = 0.38. In addition, the interaction between Target and Valence was significant, *F* (1, 24) = 12.99, *p* = 0.001, Partial η^2^ = 0.35. Further analysis showed that the accuracy rates of negative words (*M* = 0.95, *SE* = 0.01) were significantly lower than those of positive words (*M* = 0.96, *SE* = 0.01) when the targets were social words (*p* = 0.024, Parital η^2^ = 0.20), while the accuracy rates of negative words (*M* = 0.98, *SE* = 0.004) were significantly higher than those of positive words (*M* = 0.96, *SE* = 0.01) when the targets were nonsocial words (*p* = 0.013, Parital η^2^ = 0.23). Other main effect and interactions were not significant, *p*s > 0.05.

#### ERP results

##### P2 component (150 ~ 220 ms)

**Fig. 2:**
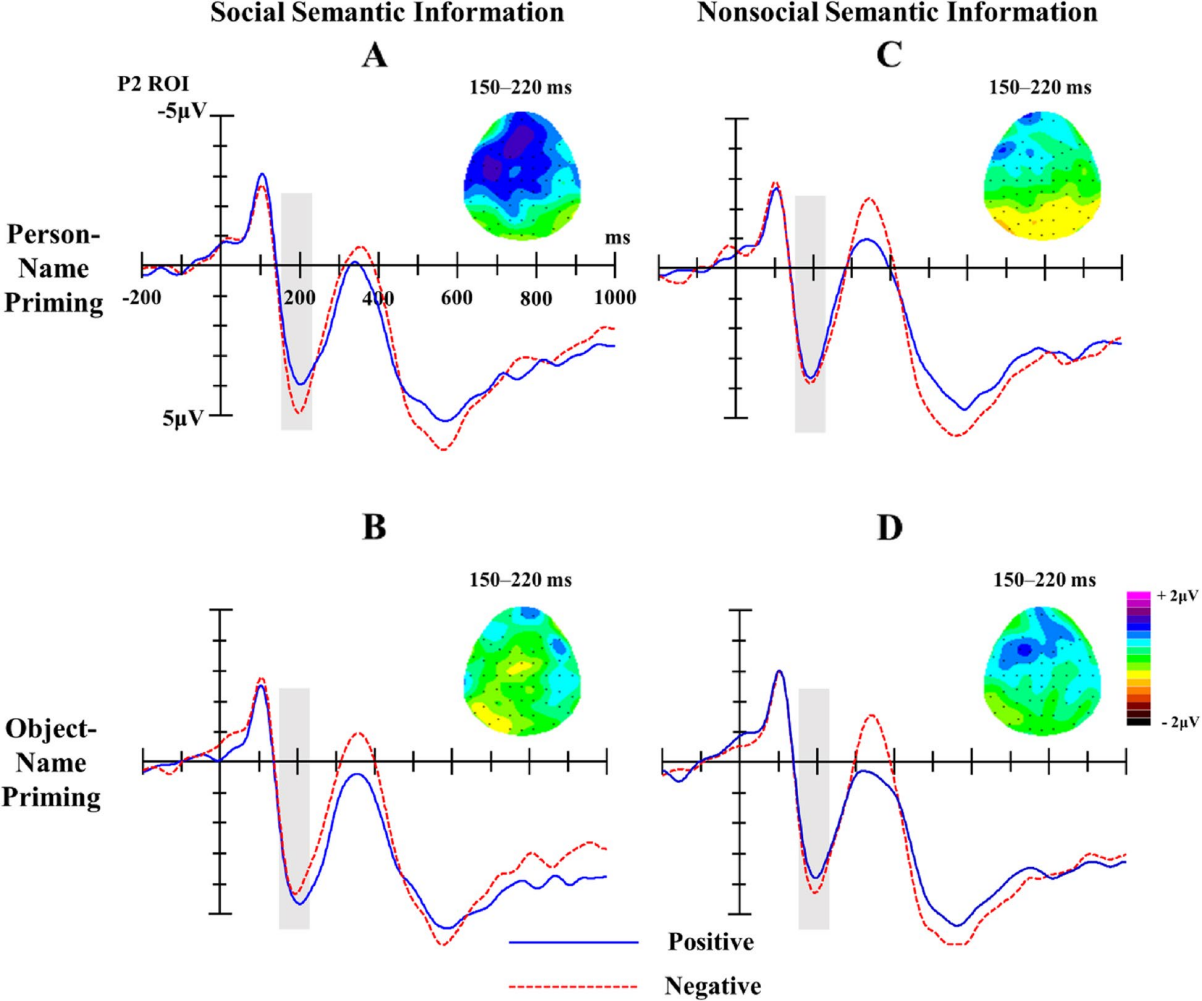
Waveforms and topographic maps induced by social semantic information (**A**, **B**) and nonsocial semantic information (**C**, **D**) with different valence after person and object-name priming; the shaded areas mark P2 components. Topographies of difference between ERPs for Positive and Negative conditions (negative minus positive trials) in the P2 time-window (150–220 ms)

The ANOVA results of P2 found that the significant main effect of Target was significant, *F* (1, 24) = 7.21, *p* = 0.013, Partial η^2^ = 0.23, with social words (*M* = 3.97 µV, *SE* = 0.56) eliciting a larger P2 amplitude than nonsocial words (*M* = 3.54 µV, *SE* = 0.60). The interaction of Target, Prime and Valence was marginally significant, *F* (1, 24) = 4.24, *p* = 0.051, Partial η^2^ = 0.15. Further analysis (see Table 2; Fig. 2) showed that for social semantic information, negative words induced a larger P2 amplitude than positive words after person-name priming, *p* = 0.016, Partial η^2^ = 0.22, while there was no difference after object-name priming, *p* = 0.431; for nonsocial semantic information, there was no difference between negative and positive words, whether after person-name priming or object-name priming, *p*s > 0.05. The other main effect and interactions were not significant, *p*s > 0.05.

**Table 2:** Mean P2 amplitudes (µV) and standard error (*SE*) across different conditions

Target	Prime	Valence	
		Positive	Negative
Social semantic information	Person names	3.488 (0.555)	4.341 (0.603)
	Object names	4.153 (0.586)	3.890 (0.644)
Nonsocial semantic information	Person names	3.331 (0.675)	3.527 (0.602)
	Object names	3.427 (0.654)	3.875 (0.621)

##### N400 component (300 ~ 400 ms)

The ANOVA results of N400 found that the significant main effects of Target [*F* (1, 24) = 6.11, *p* = 0.021, Partial η^2^ = 0.20], Prime [*F* (1, 24) = 6.31, *p* = 0.019, Partial η^2^ = 0.21], and Valence [*F* (1, 24) = 22.35, *p* < 0.001, Partial η^2^ = 0.48] were all significant. In addition, the interaction between Target and Prime was significant, *F* (1, 24) = 6.47, *p* = 0.018, Partial η^2^ = 0.21, as was the interaction between Target and Valence, *F* (1, 24) = 4.00, *p* = 0.057, Partial η^2^ = 0.14. The other interactions were not significant, *p*s > 0.05.

**Fig. 3:**
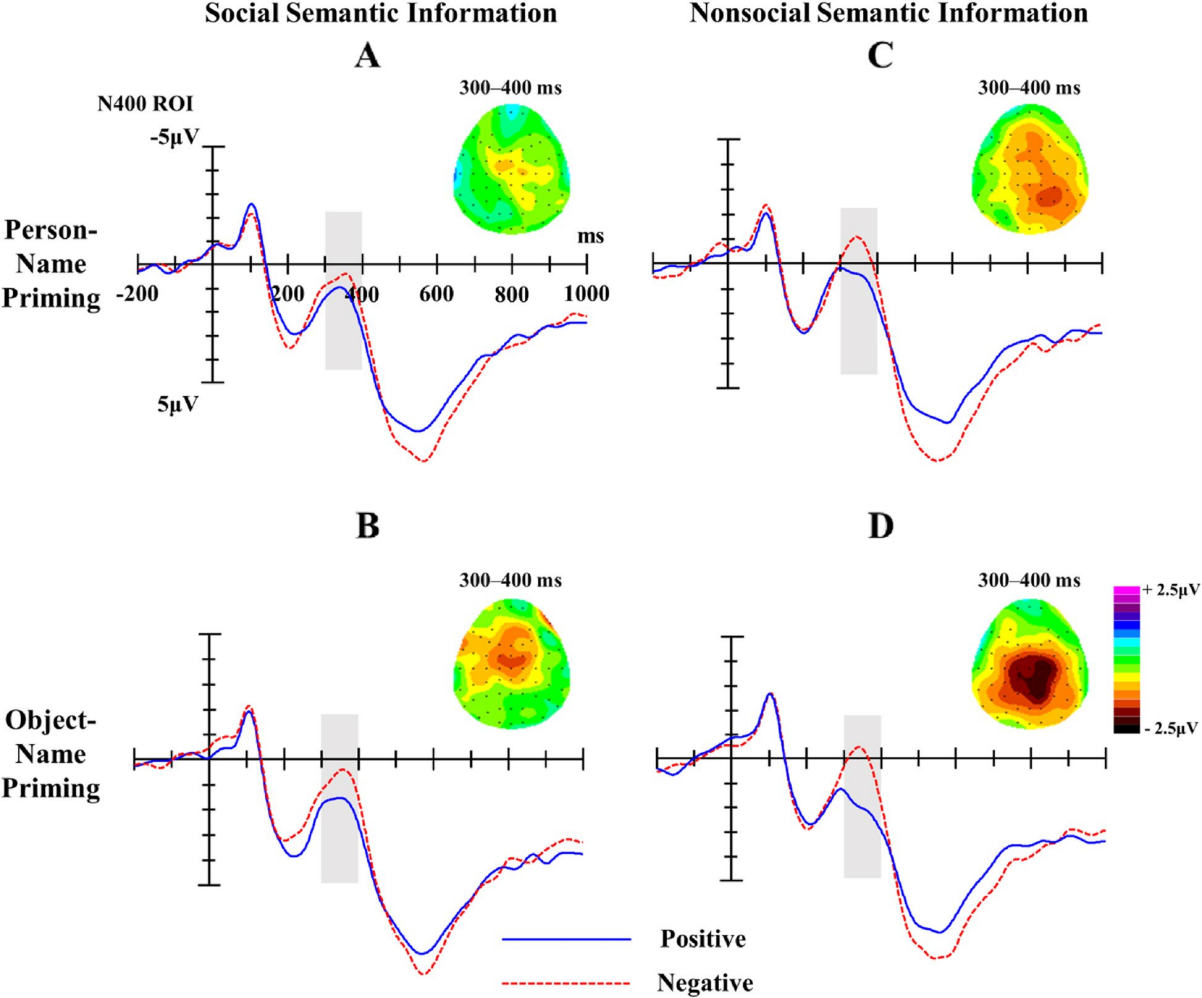
Waveforms and topographic maps induced by social semantic information (**A**, **B**) and nonsocial semantic information (**C**, **D**) with different valence after person and object-name priming; the shaded areas mark N400 components. Topographies of difference between ERPs for Positive and Negative conditions (negative minus positive trials) in the N400 time-window (300–400 ms)

The simple effect analysis of Target and Valence showed that the N400 amplitude induced by negative words was significantly larger than that induced by positive words, whether for social semantic information (*M*_Positive_ = 1.53 µV, *SE* = 0.83, *M*_Negative_ = 0.73 µV, *SE* = 0.91, *p* = 0.010, Parital η^2^ = 0.25) or nonsocial semantic information (*M*_Positive_ = 1.32 µV, *SE* = 0.79, *M*_Negative_ = − 0.29 µV, *SE* = 0.78, *p* < 0.001, Parital η^2^ = 0.46) (see Table 3; Fig. 3).

The simple effect analysis of Target and Prime showed that the N400 amplitudes induced by social words after person-name priming (*M* = 1.06 µV, *SE* = 0.87) and by social words after object-name priming (*M* = 1.20 µV, *SE* = 0.88) had no significant differences (*p* = 0.652); but the N400 amplitude induced by nonsocial words after person-name priming (*M* = − 0.04 µV, *SE* = 0.78) was significantly larger than that induced by nonsocial words after object-name priming (*M* = 1.06 µV, *SE* = 0.79, *p* = 0.002, Parital η^2^ = 0.34).

**Table 3:** Mean N400 amplitudes (µV) and standard error (*SE*) across different conditions

Target	Prime	Valence	
		Positive	Negative
Social semantic information	Person names	1.378(0.846)	0.741(0.933)
	Object names	1.690(0.860)	0.713(0.948)
Nonsocial semantic information	Person names	0.559(0.814)	–0.634(0.788)
	Object names	2.071(0.810)	0.056(0.834)

##### LPP component (450 ~ 600 ms)

The ANOVA results of LPP only found the main effect of Valence was significant, *F* (1, 24) = 14.17, *p* = 0.001, Partial η^2^ = 0.37, indicating that the negative words (*M* = 7.93 µV, *SE* = 0.81) induced larger LPP amplitudes compared to positive words (*M* = 6.70 µV, *SE* = 0.68), *p* = 0.001. The other main effect and interactions were not significant, *p*s > 0.05.

### Onset latency of negative bias effect

To investigate the exact onset of the negative bias effect, the onset latencies of the P2, N400, and LPP difference waves (i.e., the ERP amplitude induced by negative words minus the ERP amplitude induced by positive words after person/object-name priming for social and nonsocial semantic information) was estimated using the procedure outlined in Li, Hagoort, and Yang [43], which has also been used in a number of other studies [14, 44, 45].

Specifically, for the P2 difference wave of social words after person-name priming, the mean amplitudes for ROI were calculated with a step size of 10 ms latency ranges (bins) from 150 ms after target word onset. Then, the value for each latency bin was subjected to a one-sample *t* test that tested against the null hypothesis of the negative bias effect being less than zero (one-tailed t-test). The onset latency of the negative bias effect was defined as the point at which five consecutive *t*-tests yielded significant results (*p* < 0.05, in the same direction). The onset latencies of other instances were measured and determined in the same way: the onset latency of N400 or LPP negative bias from 300 or 450 ms after target word onset. Table 4 showed the results for the statistical analysis of onset latency.

**Table 4:** Onset latencies of the negative bias effect on social/nonsocial semantic information after person-name priming and object-name priming

Target	Prime	ERPs	Temporal windows (*p*)				
Social semantic information	Person names	P2	150–160 ms (0.088)	**160–170** ms (0.033)	(*p*s < 0.05)	210–220 ms (0.035)	220–230 ms (0.177)
	Object names	N400	330–340 ms (0.054)	**340–350** ms (0.024)	(*p*s < 0.05)	400–410 ms (0.037)	410–420 ms (0.142)
	Person names	LPP	490–500 ms (0.054)	**500–510** ms (0.042)	(*p*s < 0.05)	730–740 ms (0.049)	740–750 ms (0.284)
	Object names	LPP	550–560 ms (0.051)	**560–570** ms (0.031)	(*p*s < 0.05)	600–610 ms (0.034)	610–620 ms (0.063)
Nonsocial semantic information	Person names	N400	290–300 ms (0.196)	**300–310** ms (0.020)	(*p*s < 0.05)	390–400 ms (0.046)	400–410 ms (0.202)
	Object names	N400	310–320 ms (0.102)	**320–330** ms (0.012)	(*p*s < 0.05)	390–400 ms (0.031)	400–410 ms (0.054)
	Person names	LPP	500–510 ms (0.087)	**510–520** ms (0.043)	(*p*s < 0.05)	730–740 ms (0.017)	740–750 ms (0.051)
	Object names	LPP	470–480 ms (0.055)	**480–490** ms (0.046)	(*p*s < 0.05)	660–670 ms (0.050)	670–680 ms (0.138)

**Fig. 4:**
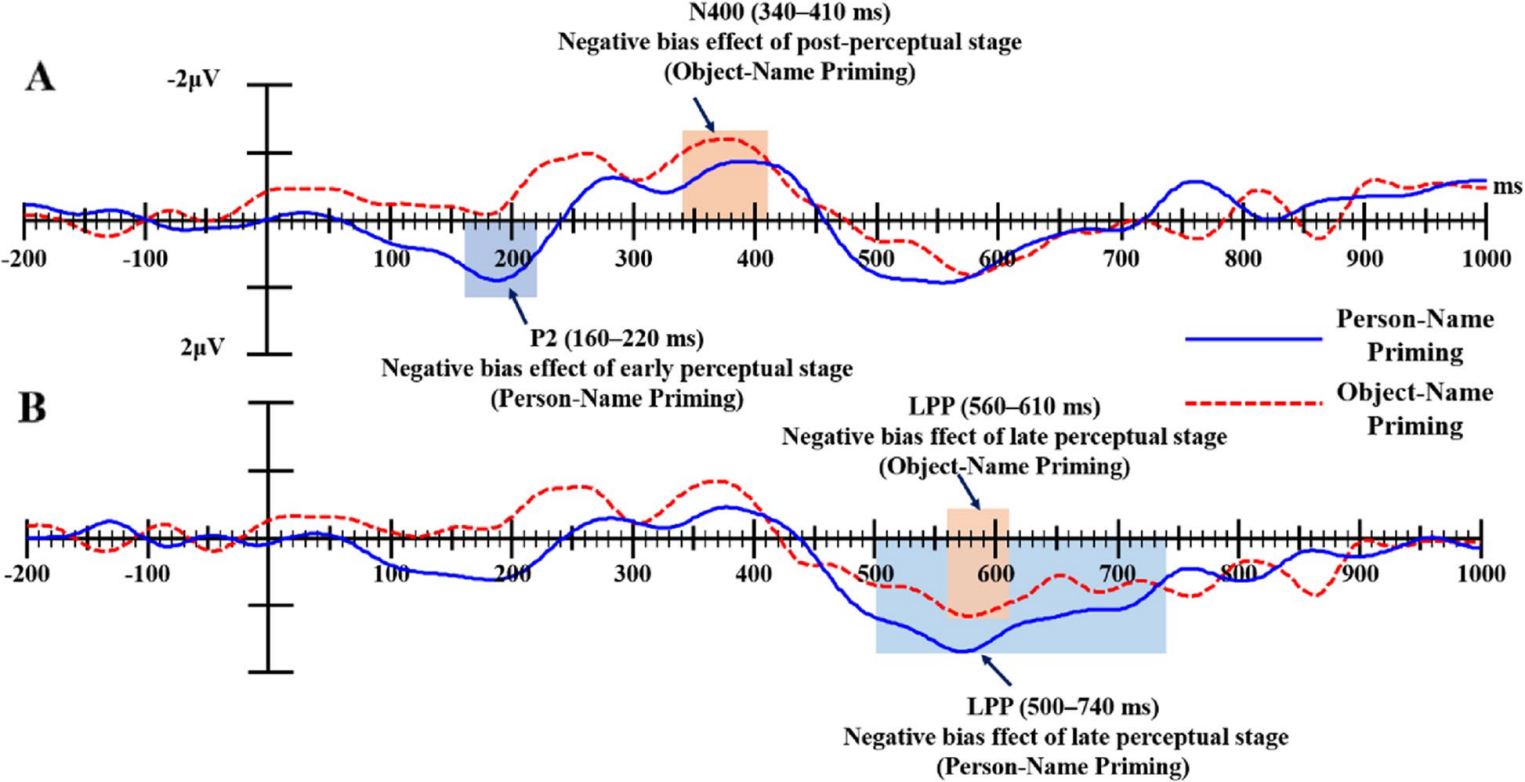
Difference waves of social semantic information after Person-Name Priming and Object-Name Priming (the ERP amplitudes elicited by negative trials minus the ERP amplitudes elicited by positive trials within each priming category)

The results found that: (1) For the social semantic information, the onset of negative bias after person-name priming started 180ms earlier than that after object-name priming (160 ms vs. 340 ms after targets emerging) in the early (P2)/post-perceptual (N400) processing stage (see Fig. 4A). Similarly, the onset of LPP negative bias after person-name priming started 60ms earlier than that after object-name priming (500 ms vs. 560 ms after targets emerging, see Fig. 4B).

**Fig. 5:**
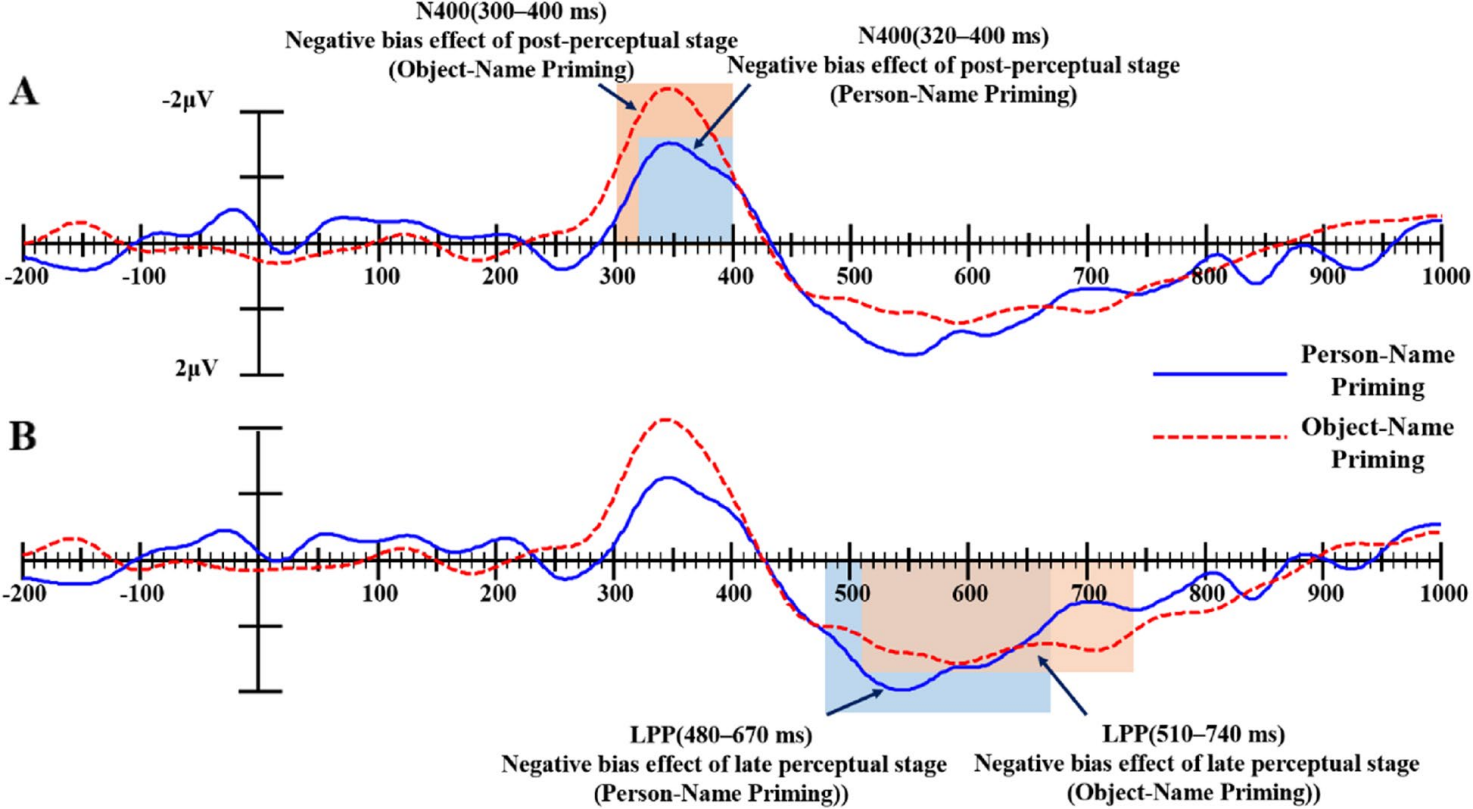
Difference waves of nonsocial semantic information after Person-Name Priming and Object-Name Priming (the ERP amplitudes elicited by negative trials minus the ERP amplitudes elicited by positive trials within each priming category)

 (2) For the nonsocial semantic information, the negative bias effect after object-name priming appeared earlier than that after person-name priming for nonsocial semantic information (see Fig. 5A), the difference is only 20 ms (300 vs. 320 ms after targets emerging). Similarly, the onset of LPP negative bias after person-name priming started 30ms earlier than that after object-name priming (480 ms vs. 510 ms after targets emerging, see Fig. 5B).

## Discussion

The present study aimed to investigate whether there is a negative bias and its specificity in the processing of social semantic information compared with nonsocial semantic information. The behavioral results showed that the response times for negative words were significantly slower than those for positive words whether for social or nonsocial semantic information and the response times for social words were slower than those for nonsocial words. In addition, the accuracy rates of negative words were significantly lower than those of positive words when the targets were social words, but there was a opposite processing pattern when the targets were nonsocial words. The ERP results found that there was a negative bias effect in the whole-time course of brain neural activity for the two types of information; the P2, N400 and LPP amplitudes induced by the negative words were larger than those induced by the positive words in the same conditions. More importantly, this effect had different characteristics in the time course for the two types of information. The negative bias effect of social semantic information started in the early perceptual stage and was significantly affected by prime type, but the negative bias effect of nonsocial semantic information started in the post-perceptual stage and was not affected by prime type which was further confirmed by investigating the exact onset of the negative bias effect. In addition, the nonsocial semantic information after person-name priming induced a larger N400 amplitude than that after object-name priming. The main findings are discussed below.

The behavioral results found the same response time pattern for the social and nonsocial semantic information. The response times for negative words were significantly slower than those for positive words whether the targets comprised social semantic information describing people or nonsocial semantic information describing objects. This result may indicate that people always expect good things, whether in terms of people or objects, while negative things are not expected, so people need more time to process negative information [17]. The accuracy results showed that there were different processing patterns for the two types of information. The accuracy of negative words was significantly lower than that of positive words when the target comprised social semantic information, while the accuracies of negative words were significantly higher than those of positive words when the targets were nonsocial semantic information. That is, participants were more likely to judge words that described people negatively as positive. According to Sears [36], individuals tend to make positive evaluations of others, so participants are more likely to make mistakes when the targets are negative words describing people.

The ERP results found that there was a negative bias in the time course of brain neural activity no matter whether the target words comprised social semantic information describing people or nonsocial semantic information describing objects, which verified the processing advantage of negative information, finding that “bad is stronger than good” [17, 46]. Specifically, the results first showed that in the early perceptual stage, negative words describing people after person-name priming induced a larger P2 amplitude than positive words, which is consistent with previous studies. Studies using words as experimental materials also found that negative stimuli induced a larger P2 amplitude than positive stimuli [23, 30, 47]. Researchers have argued that P2 is relevant to attention and its peak is over the frontal and occipito-temporal sites between 200 and 250 ms; the amplitude difference of P2 in this temporal window indicates that the negative bias occurs in the early perceptual stage [48]. The negative bias (P2) of social semantic information found in this study was also mainly distributed in the frontal scalp, and its amplitude reached its maximum at 200 ms, which may indicate that negative words describing people first capture attention in the early perceptual stage. This early negative bias may reflect an evolutionary adaptation, since rapid detection of negative stimuli is critical for individuals’ survival [46].

In addition, this study found the negative words induced a larger N400 amplitude than the positive words, that is, there was a negative bias effect whether for social semantic information or nonsocial semantic information. Previous studies found that the N400 amplitude was affected by the stimulus valence [49, 50]. For example, De Pascalis et al. [49] demonstrated a significant increase in N400 amplitude induced by negative words compared to positive and neutral words embedded in sentences. The researchers argued that this N400 negative bias effect can be explained by the theory where by N400 reflects the distribution of attention, that is, stimuli that receive more attention induce a larger N400 amplitude [43, 51, 52]. Thus, the N400 negative bias effect found in the current study can be explained by negative words attracting more attention than positive words in the post-perceptual stage.

Corresponding to the P2/N400 negative bias effect, the results showed that in the late perceptual stage, negative words induced a larger LPP amplitude than positive words in the same condition for both social semantic information and nonsocial semantic information. The LPP is a late positive component and has been shown to increase in amplitude at central-parietal electrode sites in participants viewing emotional stimuli; it usually appears about 500 ms after the presentation of stimuli and generally lasts about 300 ms [53]. Previous studies have found that emotional words [54, 55], images [56, 57], and facial expressions [58, 59] induced larger LPP amplitudes. LPP reflects the process of motivated attention, which is induced by stimuli that can trigger approach or avoidance response motivation. The increase in LPP amplitude induced by emotional stimuli indicates the increase of attentional resources allocated to such stimuli and the enhancement of response motivation [60, 61]. For example, Schupp et al. [59] found that angry faces elicited a larger LPP amplitude than happy and neutral faces, suggesting that because threatening stimuli such as angry faces are important to individual survival, more motivational attention (avoidance) was allocated to the stimuli transmitting threatening information. It may be the case that regardless of whether the words are used for describing people or objects, the negative words are more threatening and more important to individuals’ survival, so the response motivation (avoidance) and attentional resources allocated to negative words increase.

Based on fMRI technology, the researchers found that the human brain always was more active by social information compared with nonsocial information [3–6, 8, 13]. Similarly, the studies based on ERPs technology found that the social information captured more attention compared with nonsocial information [14–16]. That is, the human is highly sensitive to social information [1, 2]. The present study also verified this point. Specifically, only the negative social words induced larger P2 amplitude. In the post perceptual stage and the late perceptual stage, the negative information captured more attention than positive information. Specifically, both the negative words induced larger N400 and LPP amplitudes whether for social and nonsocial information. These results suggest that, people can rapidly discriminate the social and nonsocial information in the early perceptual stage, and then focus their attention on the negative stimuli.

The ERP results found that there was a negative bias effect on the processing of both types of information, but more importantly revealed a difference in time course between the two types of information. The most significant factor was that the negative bias effect of social semantic information obviously started earlier. The negative bias of social semantic information appeared in the early perceptual stage P2, while the negative bias of nonsocial semantic information appeared in the post-perceptual processing stage N400. This result suggests that the social information related to people is more important for social communication and even survival. The ability to distinguish positive and negative information more quickly and accurately means that a person can quickly judge whether others are friends or enemies, so as to decide whether to communicate with them or avoid them. Therefore, the P2 found in this study marked the negative bias of social semantic information in the early perceptual stage from the perspective of survival. Negative stimuli can arouse more attentional resources than positive stimuli, which reflects an automatic self-protection mechanism formed in the long-term evolution of individuals. Hence, this study found that the onset time of the negative bias effect for social semantic information occurs significantly earlier than that of nonsocial semantic information, providing evidence that the negative bias effect of social semantic information has a temporal advantage.

Another difference was that the onset latency of negative bias effect was affected by the Prime condition when the targets comprised social semantic information; the negative bias effect after person-name priming occurred earlier than that after object-name priming. However, the onset latency of the negative bias effect was not affected by the Prime condition when the targets comprised nonsocial semantic information. Specifically, the negative bias after person-name priming for social semantic information first appeared in the early perceptual stage (P2), and negative bias after object-name priming first appeared in the post-perceptual stage (N400). The LPP negative bias effect in the late perceptual stage after person-name priming was also 60ms earlier than that after object-name priming. However, for nonsocial semantic information, the onset latency of the negative bias effect after person-name or object-name priming was basically same whether in the post-perceptual stage N400 or in the late perceptual stage LPP. To sum up, the ERP results provide evidence that “person name” can promote cognitive processing about person-related information. Since this study aimed to explore the specificity of words which describe people, an important piece of social semantic information, the possible influence of a person’s name was not considered when the hypothesis was proposed. In fact, “person name” is also a very common and important piece of social information related to people. This finding is consistent with several previous studies using similar paradigms, where social information related to people can facilitate the processing of target information when used as a priming stimulus [14, 16].

In addition, another finding in this study, that nonsocial semantic information after person-name priming induced a larger N400 amplitude compared with object-name priming, also supports the idea that person-name priming promotes processing. Taking person name and object name as social and nonsocial priming categories, respectively, this study found that there was a significant N400 conflict effect for nonsocial semantic information after person-name priming, while social semantic information after object-name priming did not show a significant N400 conflict effect. In other words, compared with words used for people after object-name priming, words used for objects after person-name priming are more likely to cause a cognitive processing conflict. This may be due to “person name” being a special and important piece of social semantic information. A person name, compared with an object name, will attract more attention; this means that the brain is more sensitive to detecting whether the following target word matches it.

There are some limitations that should be taken into consideration in subsequent studies. The present study takes “person name” and “object name” as prime stimuli which has some differences between these two stimuli at the conceptual level. For example, nonsocial semantic information such as “apple” and “glove” belong to general superior concepts, but social semantic information such as “Li Ming” and “Zhao Yang” belong to specific subordinate concepts. Furthermore, it’s difficult for participants to predict the following target word (e.g., generous/fierce) after seeing a person prime (e.g., Li Ming). However, after seeing an object name (e.g., dumpling), participants were much more easily to predict the following target word (e.g., tasty/nasty). Therefore, it is necessary to set a “no priming” condition or other types of social semantic information to further verify the conclusions. In addition, the findings in the present study are only based on normal college students. Further research is needed to ascertain whether the conclusions about the presence of an early negative bias effect and the specificity of social semantic information, can be extended to individuals with mood disorders (e.g., social phobia, autism, depression, etc.).

## Conclusion

The present study revealed the existence of early negativity bias of social information and provided new evidence for the specificity of social information. The findings suggest that: (1) There is a negative bias in the processing of social semantic information and nonsocial semantic information during the whole-time course of neural activity. More importantly, the negative bias of social semantic information occurs in the early perceptual processing stage, which is significantly earlier than the onset of negative bias of nonsocial semantic information; (2) The negative bias of social semantic information is affected by priming, indicating that the words used for people after person-name priming are preferentially processed; (3) There is a significant semantic conflict N400 effect only for nonsocial semantic information.

### Supplementary Information

**Additional file 1: Appendix 1.** The Primes and Targets stimuli across different experiment conditions (Partial).
