# Supplementary material for: The early negative bias of social semantics: evidence from behavioral and ERP studies
Source: BMC Psychol. 2023 Aug 26;11:249. doi: 10.1186/s40359-023-01286-0 (PMC10464141; doi:10.1186/s40359-023-01286-0)
Supplement: Supplementary file 1 — Additional file 1: Appendix 1. The Primes and Targets stimuli across different experiment conditions (Partial). [file 40359_2023_1286_MOESM1_ESM.docx]

**Appendix 1 The Primes and Targets stimuli across different experiment conditions (Partial)**

| Target | Prime | | | |
| --- | --- | --- | --- | --- |
|  | Person Names | | Object Names | |
|  | Positive | Negative | Positive | Negative |
| Social Semantic Information | Li Ming-generous  Zhao Kai-just  Zhu Yong-open-minded  Yang Yi-amiable | Liu Rui-fierce  Gao Xiang-impolite  Li Hao-vulgar  Fang Li-arrogant | paddy-generous  dumpling-just  air-open-minded  spring water-amiable | refrigerator-fierce  headlights-impolite  computer-vulgar  desert-arrogant |
| Nonsocial Semantic Information | Li Ming-prolific  Zhao Kai-tasty  Zhu Yong-pure  Yang Xi-sweet | Liu Rui-aging  Gao Xiang-dazzling  Li Haor-radiant  Fang Li-barren | paddy-prolific  dumpling-tasty  air-pure  spring water-sweet | refrigerator-aging  headlights-dazzling  computer-radiant  desert-barren |
